# Joint Analysis of Strain and Parent-of-Origin Effects for Recombinant Inbred Intercrosses Generated from Multiparent Populations with the Collaborative Cross as an Example

**DOI:** 10.1534/g3.117.300483

**Published:** 2017-12-18

**Authors:** Yanyan Liu, Sican Xiong, Wei Sun, Fei Zou

**Affiliations:** * School of Mathematics and Statistics, Wuhan University, Hubei 430072, China; † Public Health Sciences Division, Fred Hutchinson Cancer Research Center, Seattle, Washington 98109; ‡ Department of Biostatistics, University of North Carolina at Chapel Hill, North Carolina 27599

**Keywords:** imprinting, Bayesian variable selection, parameter-expanded Gaussian priors, Multiparental Populations, MPP

## Abstract

Multiparent populations (MPP) have become popular resources for complex trait mapping because of their wider allelic diversity and larger population size compared with traditional two-way recombinant inbred (RI) strains. In mice, the collaborative cross (CC) is one of the most popular MPP and is derived from eight genetically diverse inbred founder strains. The strategy of generating RI intercrosses (RIX) from MPP in general and from the CC in particular can produce a large number of completely reproducible heterozygote genomes that better represent the (outbred) human population. Since both maternal and paternal haplotypes of each RIX are readily available, RIX is a powerful resource for studying both standing genetic and epigenetic variations of complex traits, in particular, the parent-of-origin (PoO) effects, which are important contributors to many complex traits. Furthermore, most complex traits are affected by >1 genes, where multiple quantitative trait locus mapping could be more advantageous. In this paper, for MPP-RIX data but taking CC-RIX as a working example, we propose a general Bayesian variable selection procedure to simultaneously search for multiple genes with founder allelic effects and PoO effects. The proposed model respects the complex relationship among RIX samples, and the performance of the proposed method is examined by extensive simulations.

Most human genes have functional mouse counterparts, and genomes of both organisms are organized similarly. Thus, mouse serves as a good model organism for complex human diseases. Recombinant inbred (RI) mice are among the major mouse resources in biomedical and genetic research. However, traditional mouse RI lines are derived from only two inbred parental strains, with limited a number of lines available, resulting in a low percentage (15%) of genetic variation across all mouse inbred strains (Yuan *et al.* 2011) and extensive blind spots where a sizable proportion of the genome is identical by descent. These limitations make traditional RI insufficient for studying complex traits. With the need for more powerful resources, multiparent populations (MPP) (de Koning and McIntyre 2017), a set of inbred lines using multiple lines as founders, can overcome the limitations of traditional RI lines and have become an innovative tool for fine quantitative trait locus (QTL) mapping. In the past 15 yr, different kinds of MPP have been established in plants and animals, such as nested association mapping design (Yu *et al.* 2008) and multiparent advanced generation intercross (MAGIC) populations (Cavanagh *et al.* 2008; Kover *et al.* 2009; Huang *et al.* 2015; Ladejobi *et al.* 2016) in plants, and the *Drosophila* Synthetic Population Resource (King *et al.* 2012) in animals. The mouse MPP include the collaborative cross (CC) (Complex Trait Consortium 2004), in which a genetically diverse set of eight inbred strains were selected as breeding founders (Iraqi *et al.* 2008) to generate a large number of RI lines. The eight founder strains were predicted to represent ∼90% of the genetic variation presented in laboratory mice (Threadgill and Churchill 2012). The CC thus greatly overcomes the limitations of the traditional mouse RI lines, and is the only mammalian resource with high genome-wide genetic variation that is uniformly distributed across a large, heterogeneous, and infinitely reproducible population (Threadgill *et al.* 2011).

Through the generation of RI intercrosses (RIX) of RI lines, a large number of potential “outbred” RIX samples can be generated. That is, given *L* RI lines, L(L−1) or L(L−1)/2 genetically distinct reciprocal or nonreciprocal $F_{1}$ individuals, or RIX, can be produced. An example of using CC-RIX to illustrate a distinct response to Ebola virus infection, by Rasmussen *et al.* (2014), was summarized by the editor: “the CC-RIX mice could prove valuable for preliminary screens of candidate therapeutics and vaccines.” Since all parental RI lines are isogenic at each locus, genotypes of RIX can be imputed in advance from those of their parental RI lines. Additional advantages of RIX can be found in Threadgill and Churchill (2012), in particular, its power in support of analysis of parent-of-origin (PoO) effects, where effects of certain alleles are different depending on whether those alleles are inherited maternally or paternally. PoO makes a significant contribution to the heritability of most complex traits (Mott *et al.* 2014). In addition, genomic PoO effects provide a great model to study epigenetic regulation of gene expression (Barlow 2011).

During the past 20 yr, QTL mapping methods, including analysis of variance (ANOVA), interval mapping (Lander and Botstein 1989), composite interval mapping (Jansen and Stam 1994; Zeng 1994), and multiple interval mapping (Kao and Zeng 1997; Kao *et al.* 1999), have been well developed using experimental crosses, such as backcross, $F_{2}$, and RI (see Broman (2001) for reviews), and many excellent open software packages, such as QTLCart (Basten *et al.* 1999), MapManager (Manly and Olson 1999), and R/qtl (Broman *et al.* 2003), are freely available online. For diallel data obtained from the CC founder strains, Lenarcic *et al.* (2012) employed a general Bayesian model for decomposing phenotypic variance into biologically intuitive components. Crowley *et al.* (2014) also applied a Bayesian method to a diallel cross of the eight founder strains to estimate genome-wide genetic and PoO effects (not QTL mapping) on responses to haloperidol, an antipsychotic drug. Zhang *et al.* (2014) proposed a general single QTL Bayesian framework for MAGIC data to coherently estimate haplotype and diplotype effects of founder alleles. Similarly, for MAGIC data, Wei and Xu (2016) developed a single QTL mapping method with a random-effects model by treating the founder allelic effects of each locus as random, and scanning the entire genome one locus at a time using a likelihood ratio test. For RIX mice, a mixed-effects model was developed for modeling the unbalanced relatedness among them (Tsaih *et al.* 2005; Zou *et al.* 2005). Gong and Zou (2012) proposed a more flexible, nonparametric single QTL mapping method for detecting QTL with time-varying coefficients. Hallin *et al.* (2016) applied a random-effects model to map single QTL by partitioning the variance of growth traits across different environments of yeast strains into additive, dominance, and pairwise epistatic components. However, many complex traits are affected by >1 gene, and multiple QTL mapping may be more powerful than locus-by-locus analysis. For CC-RIX data, Yuan *et al.* (2011) constructed a mixed-effects model to simultaneously map multiple QTL, but treated the eight CC founder alleles as standard biallelic single-nucleotide polymorphisms (SNPs). For many complex traits, it is arguable that modeling the effects of the eight founder alleles could lead to improved mapping power (Collaborative Cross Consortium 2012; Vered *et al.* 2014). In addition, for complex traits where PoO effects are suspected, modeling such effects may further improve QTL mapping power (Lawson *et al.* 2013) and understanding of etiologies of complex traits (Threadgill and Churchill 2012).

In this paper, for MPP-RIX data, we develop a Bayesian variable selection procedure to simultaneously map multiple genes with founder allelic effects and PoO effects. We demonstrate our method with CC-RIX data, but the method is general enough for other MPP-RIX populations. We place parameter-expanded Gaussian (PeG) priors on both the random founder allelic effects and PoO effects for variable selection.

The paper is organized as follows. In the *Statistical Method* section, we first introduce the CC-RIX experiment, then propose a random-effects model and describe a Bayesian variable selection procedure. In the *Simulation Study* section, we perform extensive simulation to examine the proposed method. Summary comments are given in the *Discussion* section.

## Statistical Method

The CC-RIX panel is the RI intercross of CC lines. For *L* CC RI lines, a total of L(L−1) reciprocal CC-RIX and L(L−1)/2 nonreciprocal CC-RIX can be generated [see Figure 1 of Zou *et al.* (2005), Yuan *et al.* (2011)]. Let *n* be the total number of CC-RIX samples and *p* be the total number of genetic markers. Further, let the phenotype of individual *i* (the dependent variable) be $y_{i}\;\left(i=1,\cdots ,n\right).$

**Figure 1:**
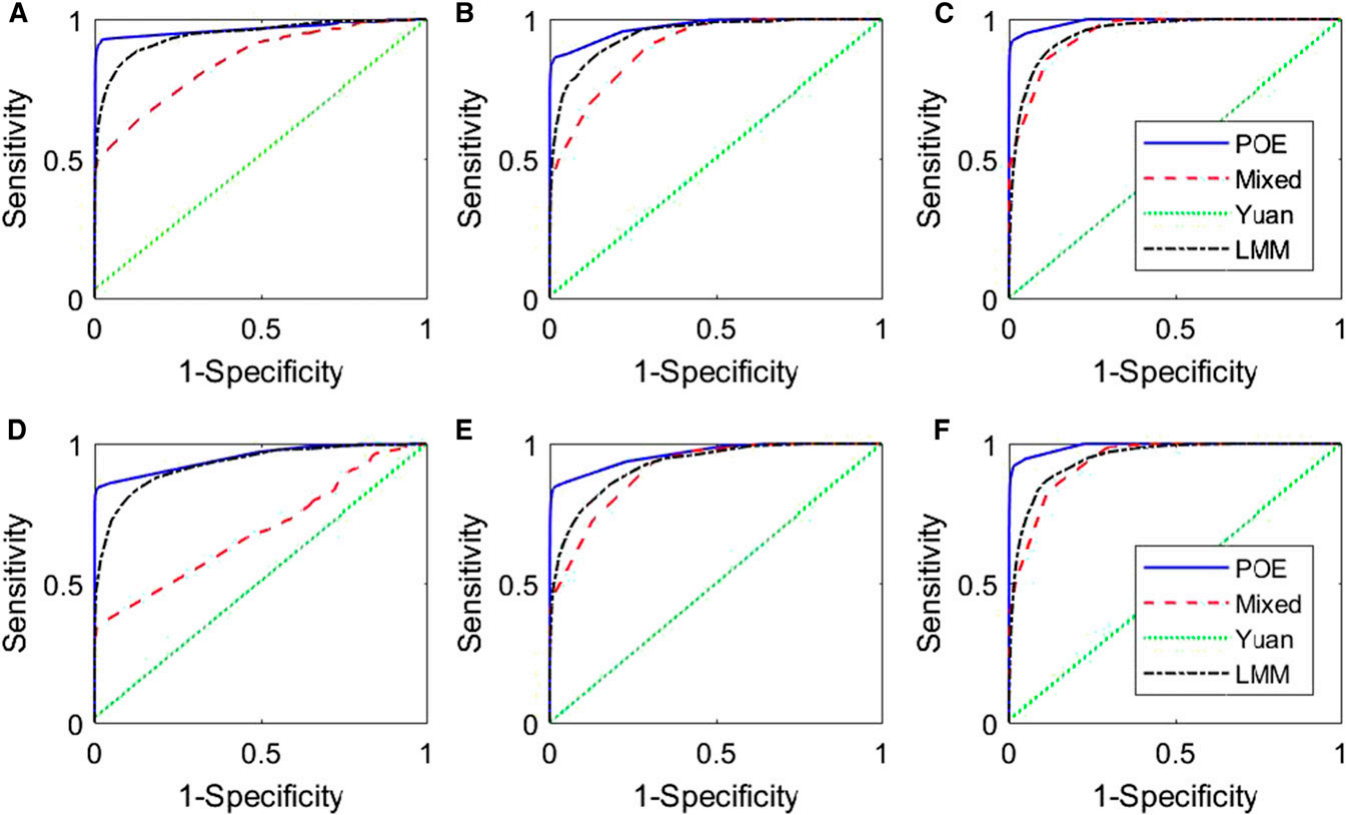
ROC curves. (A–C) for cases 1, 2, and 3, respectively, and (D–F) for cases $1^{\text{*}},$ $2^{\text{*}}$, and $3^{\text{*}},$ respectively. The legends “POE”, “Mixed”, “Yuan” and “LMM” represent our proposed model (2), the mixed model (3), Yuan’s model (5), and the LMM model (4), respectively.

### Model

In order to account for the unbalanced relatedness between the CC-RIX mice and model the eight founder alleles and PoO effects, we extend the mixed additive random-effects model of Gong and Zou (2012) and Yuan *et al.* (2011) as follows:

$$y_{i}=\mu +\sum_{j=1}^{p}\gamma_{Qj}x_{ij}^{T}\beta_{j}+\sum_{j=1}^{p}\gamma_{Pj}z_{ij}^{T}\xi_{j}+\sum_{l=1}^{L}a_{il}\alpha_{l}+e_{i}$$ (1)

where $\gamma_{Qj}$ and $\gamma_{Pj}$ are binary {0, 1} variables used to decide whether the *j*th QTL and PoO should be included in or excluded from the model and µ is the overall mean; $\beta_{j}={\left(\beta_{j1},\cdots ,\beta_{j8}\right)}^{T}$ and the *k*th element of $x_{ij}^{T}$ equals 2, 1, or 0, depending on whether ${\text{CC-RIX}}_{i}$ inherits 2, 1, or 0 copies of the *k*th founder allele (*k* = 1, ⋯, 8) at the *j*th candidate locus; $a_{il}=\#$ of parents of ${\text{CC-RIX}}_{i}$ that are equal to ${\text{CC-RI}}_{l}.$ Let A be an *n* × L matrix whose (i,l)th element equals $a_{il}.$ Clearly, $\sum_{l=1}^{L}a_{il}\equiv 2$ for all *i* = 1, 2, ⋯, *n*, since each CC-RIX has two and only two parents. Therefore, $\beta_{jk}\left(j=1,\cdots ,p\right)$ represents the *k*th founder allelic effect of locus *j*, and $\alpha_{l}\left(l=1,\cdots ,L\right)$ represents the random polygenic effect of founder strain *l*. We let $\alpha_{l}$ follow $N\left(0,\sigma_{a}^{2}\right)$ (l=1,2,⋯,L), and the random error $e_{i}$ follow $N\left(0,\sigma_{e}^{2}\right)$ (i=1,2,⋯,n).

At a given locus, PoO effects can only be estimated from individuals with heterogeneous genotypes, since for those with homozygote genotypes, the parental contributions cannot be distinguished. Label the eight-founder alleles A, B, C, D, E, F, G, and H by numbers 1–8, and let the $k^{th}$ element of $z_{ij}={\left(z_{ij1},\cdots ,z_{ij8}\right)}^{T}$ be equal to 1 or −1 if the maternal or paternal allele of the *i*th subject at the *j*th locus equals the *k*th founder allele, and 0 otherwise. For those CC-RIX samples with a homozygous genotype at a given locus, such as AA or BB, we let $z_{ij}^{\prime }=0_{1\times 8}.$ Therefore, $\xi_{j}={\left(\xi_{j1},\cdots ,\xi_{j8}\right)}^{T}$ represents the PoO effects of the *j*th locus.

Model (1) can be expressed by the following matrix form:

$$y=\mu +\sum_{j=1}^{p}\gamma_{Qj}x_{j}\beta_{j}+\sum_{j=1}^{p}\gamma_{Pj}z_{j}\xi_{j}+\mathrm{A\alpha}+e$$ (2)

where $y={\left(y_{1},\cdots ,y_{n}\right)}^{T},$ $\mu =\mu 1_{n},$ $x_{j}={\left(x_{1j},\cdots ,x_{nj}\right)}^{T},$ $z_{j}={\left(z_{1j},\cdots ,z_{nj}\right)}^{T},$ $\alpha ={\left(\alpha_{1},\cdots ,\alpha_{L}\right)}^{T},$ $e={\left(e_{1},\cdots ,e_{n}\right)}^{T},$ and $1_{n}={\left(1,\cdots ,1\right)}^{T}.$

To enable selection of QTL and PoO effects, we reexpress the eight-dimensional vectors $\beta_{j}$ and $\xi_{j}$ as the expanded parameters ${\tilde{\beta }}_{j}$ and ${\tilde{\xi }}_{j}$ for j=1,⋯,p, respectively, such that ${\tilde{\beta }}_{j}=\gamma_{Qj}\beta_{j}$ and ${\tilde{\xi }}_{j}=\gamma_{Pj}\xi_{j}.$ This parameter-expansion idea was first proposed by Kuo and Mallick (1998) and has an advantage in selecting important variables and shrinking coefficients.

In the above model (2), the observed data are y, marker genotypes $x=\left(x_{1},\cdots ,x_{p}\right),$ PoO information matrix $z=\left(z_{1},\cdots ,z_{p}\right)$, and parental CC-RI information matrix A. The unobserved variables include *μ*, $\beta ={\left(\beta_{1}^{T},\cdots ,\beta_{p}^{T}\right)}^{T},$ $\xi =\left(\xi_{1}^{T},\cdots ,\xi_{p}^{T}\right),$ α, $\sigma_{e}^{2},$ $\sigma_{a}^{2}$, and the indicators $\gamma_{Q}=\left\{\gamma_{Q1},\cdots ,\gamma_{Qp}\right\}$ and $\gamma_{P}=\left\{\gamma_{P1},\cdots ,\gamma_{Pp}\right\}$. We assign the following conjugate Gaussian priors to the $j^{th}$ QTL and $j^{th}$ PoO effects as:

$$\beta_{j}∼N_{8}\left(0,\sigma_{Qj}^{2}I_{8}\right)\;\text{and}\;\xi_{j}∼N_{8}\left(0,\sigma_{Pj}^{2}I_{8}\right)\;\text{for}\;j=1,\cdots ,p.$$

For convenience, we name the above hierarchical priors for QTL and PoO coefficients as PeG priors in the sequel. We assign conjugate noninformation hyper-priors to the variance parameters

$$P\left(\sigma_{Qj}^{2}\right)\propto \frac{1}{\sigma_{Qj}^{2}},\;P\left(\sigma_{Pj}^{2}\right)\propto \frac{1}{\sigma_{Pj}^{2}},\;P\left(\sigma_{a}^{2}\right)\propto {\left(\sigma_{a}^{2}\right)}^{\delta -1}\;\text{and}\;P\left(\sigma_{e}^{2}\right)\propto \frac{1}{\sigma_{e}^{2}}$$

where δ(0<δ≤1/2) is used to ensure that the posterior distribution is proper (ter Braak *et al.* 2005). The above PeG priors are equivalent to “spike and slab” point mass mixture Gaussian priors (Mitchell and Beauchamp 1988; George and McCulloch 1993) for the expanded parameters

$${\tilde{\beta }}_{j}∼\gamma_{Qj}\delta_{0}+\left(1-\gamma_{Qj}\right)N_{8}\left(0,\sigma_{Qj}^{2}I_{8}\right)\;\text{for}\;j=1,\cdots ,p.$$

and

$${\tilde{\xi }}_{j}∼\gamma_{Pj}\delta_{0}+\left(1-\gamma_{Pj}\right)N_{8}\left(0,\sigma_{Pj}^{2}I_{8}\right)\;\text{for}\;j=1,\cdots ,p.$$

It is well known that these priors can achieve variable selection. However, compared with mixture priors, the PeG priors can employ a block Gibbs sampler to update the two blocks of parameters, one for $\beta^{s}$ and $\xi^{s}$ (corresponding to the selected predictors) and one for $\beta^{u}$ and $\xi^{u}$ (corresponding to the unselected predictors) in turn, and therefore can dramatically reduce computational time, especially for high-dimensional data with large *p*.

For indicator variables $\gamma_{Qj}$ and $\gamma_{Pj},$ we specify Bernoulli priors with inclusion probability $0<\eta_{Qj}<1$ and $0<\eta_{Pj}<1$ for j=1,⋯,p, respectively. To be more flexible, we further apply hierarchical uniform priors to $\eta_{Qj}$ and $\eta_{Pj}:$

$$\eta_{Qj}∼U\left[0,1\right],\;\text{and}\;\eta_{Pj}∼U\left[0,1\right],\;\text{for}\;j=1,\cdots ,p.$$

Lastly, we specify a flat prior on *μ* as P(μ)∝1.

### Block Gibbs sampling algorithm for posterior computation

The specific priors above result in known marginal conditional distributions for all variables. The blockwise Gibbs sampling algorithm that we employ can be summarized as follows:

First, we initiate $\sigma_{a}^{2},$ $\sigma_{e}^{2},$ $\sigma_{Q}^{2}=\left\{\sigma_{Q1}^{2},\cdots ,\sigma_{Qp}^{2}\right\},$ $\sigma_{P}^{2}=\left\{\sigma_{P1}^{2},\cdots ,\sigma_{Pp}^{2}\right\},$ $\eta_{Q}=\left\{\eta_{Q1},\cdots ,\eta_{Qp}\right\}$, and $\eta_{P}=\left\{\eta_{P1},\cdots ,\eta_{Pp}\right\}$ from uniform distribution U(0,1), then sample other parameters β, ξ, α, and indicators $\gamma_{Q}$ and $\gamma_{P}$ from their priors. We then perform the following block Gibbs sampling procedures. Superscripts (t) and (t+1) signify the Markov chain Monte Carlo (MCMC) iterations, and t=0 refers to the initial iteration.

*Step 1*. Updating *μ*: $\mu^{\left(t+1\right)}$ is drawn from the normal distribution.
  $$N\left(\frac{1}{n}1_{n}^{\prime }\left(y-\sum_{j=1}^{p}\gamma_{Qj}^{\left(t\right)}x_{j}\beta_{j}^{\left(t\right)}-\sum_{j=1}^{p}\gamma_{Pj}^{\left(t\right)}z_{j}\xi_{j}^{\left(t\right)}-A\alpha^{\left(t\right)}\right),\frac{\sigma_{e}^{2\left(t\right)}}{n}\right)$$
*Step 2*. Updating β: we divide the long vector $\beta^{\left(t\right)}$ into two blocks $\beta^{s\left(t\right)}={\left({\left(\beta_{s_{1}}^{\left(t\right)}\right)}^{\prime },\cdots ,{\left(\beta_{s_{m_{1}}}^{\left(t\right)}\right)}^{\prime }\right)}^{\prime }$ and $\beta^{u\left(t\right)}={\left({\left(\beta_{u_{1}}^{\left(t\right)}\right)}^{\prime },\cdots ,{\left(\beta_{u_{m_{0}}}^{\left(t\right)}\right)}^{\prime }\right)}^{\prime }$ corresponding to the selected ($\gamma_{Qj}^{\left(t\right)}=1$) and unselected ($\gamma_{Qj}^{\left(t\right)}=0$) predictors, respectively. We then sample $\beta_{u_{h}}^{\left(t+1\right)}\left(h=1,\cdots ,m_{0}\right)$ from their priors, *i.e.*, the eight-dimensional multivariate normal distribution with zero mean and covariance matrix $\sigma_{Q,u_{h}}^{2\left(t\right)}I_{8},$ and sample $\beta_{s_{h}}^{\left(t+1\right)}\left(h=1,\cdots ,m_{1}\right)$ from the eight-dimensional multivariate normal distribution with mean $1/\sigma_{e}^{2\left(t\right)}\Sigma_{\beta_{s_{h}}}^{\left(t\right)}{\left(x_{s_{h}}\right)}^{\prime }\left(y-\mu^{\left(t+1\right)}1_{n}-\sum_{j<h}\gamma_{Q,s_{j}}^{\left(t\right)}x_{s_{j}}\beta_{s_{j}}^{\left(t+1\right)}-\sum_{j>h}\gamma_{Q,s_{j}}^{\left(t\right)}x_{s_{j}}\beta_{s_{j}}^{\left(t\right)}-\sum_{j=1}^{p}\gamma_{Pj}^{\left(t\right)}z_{j}\xi_{j}^{\left(t\right)}-A\alpha^{\left(t\right)}\right)$ and covariance matrix $\Sigma_{\beta_{s_{h}}}^{\left(t\right)}=\sigma_{Q,s_{h}}^{2\left(t\right)}{\left(\sigma_{Q,s_{h}}^{2\left(t\right)}/\sigma_{e}^{2\left(t\right)}{\left(x_{s_{h}}\right)}^{\prime }x_{s_{h}}+I_{8}\right)}^{-1}.$
*Step 3*. Updating ξ: similar to Step 2, we divide $\xi^{\left(t\right)}$ into two parts, $\xi^{s\left(t\right)}={\left({\left(\xi_{s_{1}}^{\left(t\right)}\right)}^{\prime },\cdots ,{\left(\xi_{s_{m_{1}}}^{\left(t\right)}\right)}^{\prime }\right)}^{\prime }$ and $\xi^{u\left(t\right)}={\left({\left(\xi_{u_{1}}^{\left(t\right)}\right)}^{\prime },\cdots ,{\left(\xi_{u_{m_{0}}}^{\left(t\right)}\right)}^{\prime }\right)}^{\prime }$ corresponding to the selected ($\gamma_{Pj}^{\left(t\right)}=1$) and unselected ($\gamma_{Pj}^{\left(t\right)}=0$) predictors, respectively. Then $\xi_{u_{h}}^{\left(t+1\right)}\left(h=1,\cdots ,m_{0}\right)$ is sampled from their priors, *i.e.*, the eight-dimensional multivariate normal distribution with zero mean and covariance matrix $\sigma_{P,uh}^{2\left(t\right)}I_{8},$ and $\xi_{s_{h}}^{\left(t+1\right)}\left(h=1,\cdots ,m_{1}\right)$ is sampled from the eight-dimensional multivariate normal distribution with mean$1/\sigma_{e}^{2\left(t\right)}\Sigma_{\xi_{s_{h}}}^{\left(t\right)}{\left(z_{s_{h}}\right)}^{\prime }\left(y-\mu^{\left(t+1\right)}1_{n}-\sum_{j=1}^{p}\gamma_{Qj}^{\left(t\right)}x_{j}\beta_{j}^{\left(t+1\right)}-\sum_{j<h}\gamma_{P,s_{j}}^{\left(t\right)}z_{s_{j}}\xi_{s_{j}}^{\left(t+1\right)}-\sum_{j>h}\gamma_{P,s_{j}}^{\left(t\right)}z_{s_{j}}\xi_{s_{j}}^{\left(t\right)}-A\alpha^{\left(t\right)}\right)$ and covariance matrix $\Sigma_{\xi_{s_{h}}}^{\left(t\right)}=\sigma_{P,s_{h}}^{2\left(t\right)}{\left(\sigma_{P,s_{h}}^{2\left(t\right)}/\sigma_{e}^{2\left(t\right)}{\left(z_{s_{h}}\right)}^{\prime }z_{s_{h}}+I_{8}\right)}^{-1}.$
*Step 4*. Updating α: $\alpha^{\left(t+1\right)}$ is drawn from the multivariate normal distribution with mean $1/\sigma_{e}^{2\left(t\right)}\Sigma_{\alpha }^{\left(t\right)}A\prime \left(y-\mu^{\left(t+1\right)}1_{n}-\sum_{j=1}^{p}\gamma_{Qj}^{\left(t\right)}x_{j}\beta_{j}^{\left(t+1\right)}-\sum_{j=1}^{p}\gamma_{Pj}^{\left(t\right)}z_{j}\xi_{j}^{\left(t+1\right)}\right)$ and covariance matrix $\Sigma_{\alpha }^{\left(t\right)}=\sigma_{a}^{2\left(t\right)}{\left(\sigma_{a}^{2\left(t\right)}/\sigma_{e}^{2\left(t\right)}A\prime A+I_{L}\right)}^{-1}.$
*Step 5*. Updating $\sigma_{Qj}^{2}\left(1\leq j\leq p\right):$ $\sigma_{Qj}^{2\left(t+1\right)}$ is sampled from the scale-inverted $\chi^{2}$ distribution, ${\left\|\beta_{j}^{\left(t+1\right)}\right\|}^{2}/\chi_{8}^{2}.$
*Step 6*. Updating $\sigma_{Pj}^{2}\left(1\leq j\leq p\right):$ $\sigma_{Pj}^{2\left(t+1\right)}$ is sampled from the scale-inverted $\chi^{2}$ distribution, ${\left\|\xi_{j}^{\left(t+1\right)}\right\|}^{2}/\chi_{8}^{2}.$
*Step 7*. Updating $\sigma_{a}^{2}:$ the random-effects variance $\sigma_{a}^{2\left(t+1\right)}$ is sampled from the scale-inverted $\chi^{2}$ distribution, ${\left\|\alpha^{\left(t+1\right)}\right\|}^{2}/\chi_{L-2\delta }^{2}.$
*Step 8*. Updating $\sigma_{e}^{2}:$ the residual variance $\sigma_{e}^{2\left(t+1\right)}$ is sampled from the scale-inverted $\chi^{2}$ distribution, ${\left\|y-\mu^{\left(t+1\right)}1_{n}-\sum_{j=1}^{p}\gamma_{Qj}^{\left(t\right)}x_{j}\beta_{j}^{\left(t+1\right)}-\sum_{j=1}^{p}\gamma_{Pj}^{\left(t\right)}z_{j}\xi_{j}^{\left(t+1\right)}-A\alpha^{\left(t+1\right)}\right\|}^{2}/\chi_{n}^{2}$
*Step 9*. Updating $\gamma_{Qj}\left(1\leq j\leq p\right):$ $\gamma_{Qj}^{\left(t+1\right)}$ is sampled from the Bernoulli distribution with success probability $p_{j}^{Q\left(t\right)}=\eta_{Qj}^{\left(t\right)}/\left(\eta_{Qj}^{\left(t\right)}+\left(1-\eta_{Qj}^{\left(t\right)}\right)p_{j0}^{Q\left(t\right)}\right),$ where $p_{j0}^{Q\left(t\right)}=\exp\left[-1/2\sigma_{e}^{2\left(t+1\right)}(2{\left(K_{j}^{Q\left(t\right)}\right)}^{\prime }x_{j}\beta_{j}^{\left(t+1\right)}-{\left(\beta_{j}^{\left(t+1\right)}\right)}^{\prime }\left(x_{j}^{\prime }x_{j}\right)\beta_{j}^{\left(t+1\right)})\right],$ and $K_{j}^{Q\left(t\right)}=y-\mu^{\left(t+1\right)}1_{n}-\sum_{k<j}\gamma_{Qk}^{\left(t+1\right)}x_{k}\beta_{k}^{\left(t+1\right)}-\sum_{k>j}\gamma_{Qk}^{\left(t\right)}x_{k}\beta_{k}^{\left(t+1\right)}-\sum_{j=1}^{p}\gamma_{Pj}^{\left(t\right)}z_{j}\xi_{j}^{\left(t+1\right)}-A\alpha^{\left(t+1\right)}.$
*Step 10*. Updating $\gamma_{Pj}\left(1\leq j\leq p\right):$ $\gamma_{Pj}^{\left(t+1\right)}$ is sampled from the Bernoulli distribution with success probability $p_{j}^{P\left(t\right)}=\eta_{Pj}^{\left(t\right)}/\left(\eta_{Pj}^{\left(t\right)}+\left(1-\eta_{Pj}^{\left(t\right)}\right)p_{j0}^{P\left(t\right)}\right),$ where $p_{j0}^{P\left(t\right)}=\exp\left[-1/2\sigma_{e}^{2\left(t+1\right)}(2{\left(K_{j}^{P\left(t\right)}\right)}^{\prime }z_{j}\xi_{j}^{\left(t+1\right)}-{\left(\xi_{j}^{\left(t+1\right)}\right)}^{\prime }\left(z_{j}^{\prime }z_{j}\right)\xi_{j}^{\left(t+1\right)})\right],$ and $K_{j}^{P\left(t\right)}=y-\mu^{\left(t+1\right)}1_{n}-\sum_{j=1}^{p}\gamma_{Qj}^{\left(t+1\right)}x_{j}\beta_{j}^{\left(t+1\right)}-\sum_{k<j}\gamma_{Pk}^{\left(t+1\right)}z_{k}\xi_{k}^{\left(t+1\right)}-\sum_{k>j}\gamma_{Pk}^{\left(t\right)}z_{k}\xi_{k}^{\left(t+1\right)}-A\alpha^{\left(t+1\right)}.$
*Step 11*. Updating $\eta_{Qj}\left(1\leq j\leq p\right):$ $\eta_{Qj}^{\left(t+1\right)}$ is sampled from the beta distribution $Beta\left(1+\gamma_{Qj}^{\left(t+1\right)},2-\gamma_{Qj}^{\left(t+1\right)}\right).$
*Step 12*. Updating $\eta_{Pj}\left(1\leq j\leq p\right):$ $\eta_{Pj}^{\left(t+1\right)}$ is sampled from the beta distribution $Beta\left(1+\gamma_{Pj}^{\left(t+1\right)},2-\gamma_{Pj}^{\left(t+1\right)}\right).$

### Data availability

Supplemental Material, File S1 contains five supplemental figures. The MATLAB code used to analyze the simulated data is provided in File S2.

## Simulation Study

In this section, we run extensive simulations to evaluate the performance of the proposed Bayesian method. We apply the loop design in Zou *et al.* (2005) and Yuan *et al.* (2011) by ordering the *L* CC-RI lines randomly and forming them into a circle, and then mating each CC-RI line (clockwise) with the next three CC-RI lines after it (*i.e.*, CC-RI_1_ mated with CC-RI_2_, CC-RI_3_, and CC-RI_4_; CC-RI_2_mated with CC-RI_3_, CC-RI${}_{4}$, and CC-RI; and so on). For L=100, in this way, we can generate a total of n=300 CC-RIX samples. Parental CC-RI genotypes are simulated in R/qtl (Broman *et al.* 2003). All the simulation results are based on total 100 replications for each simulation setup.

In model (2), we set the overall mean *μ* = 1, the variance of random errors $\sigma_{e}^{2}=1,$ the polygenic effect variance $\sigma_{a}^{2}=1$, and $\delta =10^{-3}.$ Nineteen chromosomes each with a length of 70 cM are simulated, on which total *p* = 133, 266, and 1330 evenly spaced markers (resulting in 10-, 5-, and 1-cM intervals between nearby markers on each chromosome, named cases 1–3) are generated, corresponding to three marker density cases. Among the total *p* simulated markers, we randomly select five markers, and let the first three markers have QTL effects the last three markers have PoO effects. That is, the first two genes only have QTL effects, and the last two only have PoO effects. However, the middle-selected marker has both QTL and PoO effects. The corresponding variances of QTL or PoO effects of the selected markers are all set to 1.

To better assess the performance of our method in situations where multiple nearby SNPs jointly affect the outcome, for cases 1–3, we now let the number of causal SNPs in each of the first two QTL be 2 instead of 1. Specifically, we randomly select two nearby SNPs for each of the first two simulated QTL and denote the alleles of the two SNPs *A* and *a*. Based on the haplotype frequencies $f_{AA},$ $f_{Aa},$ $f_{aA}$, and $f_{aa}$ (without loss of generality, we assume that $f_{AA}\geq f_{Aa}\geq f_{aA}\geq f_{aa}$), we create three haplotype allelic groups, where haplotype AA is group 1, Aa is group 2, and the other two are group 3, and set the genetic effects of the three groups to 1, 2, and 3, respectively. The three new simulation setups are labeled as cases 1^∗^ to 3^∗^.

For each simulated data point, we generate a single long chain with 20,000 cycles, of which the first 10,000 cycles are discarded as burn-in, resulting in a total of 10,000 samples for post-MCMC analysis. All the analysis is done in MATLAB, and the MATLAB source code is submitted as supplemental material (File S2).

For comparison, we fit each simulated data point with the following three models.

### The same model as (2), but without the PoO terms

$$y=\mu +\sum_{j=1}^{p}\gamma_{Qj}x_{j}\beta_{j}+\mathrm{A\alpha}+e$$ (3)

All the priors are set to be the same as their counterparts in model (2). For convenience, we subsequently call model (3) the “mixed model”.

### Linear mixed-effects model (LMM) for single gene mapping

$$y=x_{j}\beta_{j}+z_{j}\xi_{j}+\mathrm{A\alpha}+e,1\leq j\leq p.$$ (4)

Here, random effects $\alpha ∼N\left(0,\sigma_{a}^{2}I_{L}\right)$ and random errors $e∼N\left(0,\sigma_{e}^{2}I_{n}\right),$ and the random effects α are also assumed to be independent of the random errors e as before. Moreover, $\beta_{j}={\left(\beta_{j1},\cdots ,\beta_{j8}\right)}^{\prime }$ and $\xi_{j}={\left(\xi_{j1},\cdots ,\xi_{j8}\right)}^{\prime }$ are the fixed QTL and PoO effects, respectively, of the *j*th tested locus. Given the constraints that $\sum_{k=1}^{8}x_{ijk}=2$ and $\sum_{k=1}^{8}z_{ijk}=0$ for i=1,⋯,n,j=1,⋯,p, we force the intercept μ in model (4) to be **0** to overcome the identifiability problem, and jointly test the effects of the *j*th locus as:

$$H_{0j}:\beta_{j1}=\beta_{j2}=\cdots =\beta_{j8},\&\;\xi_{j1}=\xi_{j2}=\cdots =\xi_{j8}.$$

In contrast to *H*_1_*_j_*, some of the equations in $H_{0j}$ are not satisfied. After obtaining the maximum likelihood estimates of the parameters in model (4), we perform a test with the following log-odds ratio (LOD):

$$LOD_{j}=\log_{10}\frac{L_{1j}}{L_{0j}}$$

which is equivalent to the log-likelihood ratio test, where $L_{0j}$ and $L_{1j}$ are the likelihood of model (4) under null hypothesis $H_{0j}$ and alternative hypothesis $H_{1j},$ respectively. Since the above hypothesis test is performed *p* times, it is necessary to find an appropriate significance threshold to control the multiple testing, which can be obtained, for example, by modified permutation procedures (Zou *et al.* 2005). However, in our comparison, we evaluate the receiver operating characteristic (ROC) curve, which only requires the use of LOD scores.

### Yuan’s Bayesian method

$$y_{i}=\mu +\sum_{j=1}^{p}z_{ij}a_{j}+\sum_{l=1}^{L}a_{il}\alpha_{l}+e_{i}$$ (5)

Here, $a_{j}$ is the effect of the *j*th putative QTL with $z_{ij}=\sqrt{2}m_{ij}$, where $m_{ij}\left(i=1,\cdots ,n,j=1,\cdots ,p\right)$ equals −1, 0, or 1 if the putative QTL genotype is aa,Aa, or AA, respectively. The other parameters are set the same as those in the mixed model. The prior of $a_{j}$ is set to $N\left(0,\sigma_{j}^{2}\right),$ 1 ≤ *j* ≤ *p* in Yuan *et al.* (2011).

To compare the methods, ROC curves (Fawcett 2006), where true positive rates (also known as sensitivity) are plotted against false positive rates (also known as 1-specificity) evaluated at various threshold cut-offs. To estimate the sensitivity (the proportion of positives that are correctly identified as such) and specificity (the proportion of negatives that are correctly identified as such), we define false and true positive findings as follows. If a detected locus falls no more than 10 cM apart from any simulated genes, we call it a true positive finding, otherwise a false positive finding. Then, combining the outputs of each model for the 100 data sets, we can calculate the corresponding sensitivity and specificity. Specifically, for the *j*th marker, we record the LOD scores, $LOD_{j}$, for the LMM model; the maximum posterior frequency between $\gamma_{Qj}$ and $\gamma_{Pj}$ for our proposed method; the posterior frequency $\gamma_{Qj}$ for the mixed model; and the posterior mean of $\sigma_{j}^{2}$ for Yuan’s model. The corresponding area under the ROC curve (AUC) is also calculated for each of the four models, and the results are presented in Table 1. Generally speaking, a model with a higher AUC value indicates on average a better performance compared with those with lower AUC scores (Fawcett 2006).

**Table 1:** Simulation settings and AUC values

Case	IS*^a^*	*p*	*$AUC_{P}$**^b^*	*$AUC_{M}$**^c^*	*$AUC_{Y}$**^d^*	*$AUC_{L}$**^e^*
1	10	133	0.9642	0.8499	0.5155	0.9436
2	5	266	0.9722	0.9022	0.5029	0.9463
3	1	1330	0.9915	0.9489	0.5000	0.9525
1*	10	133	0.9499	0.6885	0.5085	0.9264
2*	5	266	0.9624	0.9056	0.4998	0.9238
3*	1	1330	0.9908	0.9393	0.5040	0.9482

a Interval space (IS; cM) between nearby markers.

b AUC values of our proposed model (2).

c AUC values of the mixed model (3).

d AUC values of Yuan’s model (5).

e AUC values of the LMM model (4).

From Table 1, it is clear that our proposed method outperforms the other three methods for all cases, regardless of whether there is a single causal SNP or multiple causal SNPs in each QTL. Yuan’s method fails in all the simulated cases, which is expected as the method only models biallelic SNP effects instead of the founder allelic effects that we simulate. The LMM model outperforms the mixed model, in particular when the marker density is sparse. This phenomenon is further confirmed by Figure 1, where the ROC curves of the proposed method are always higher than the ROC curves of the other three methods; the ROC curves of Yuan’s model fall along the 45-degree line, indicating its low power for mapping genes with founder allelic effects. The ROC curves of the LMM model fall between those of our proposed model and the mixed model in cases 1 and 2, and cases 1* and 2*, but cross with the ROC curves of the mixed model in cases 3 and 3*. Figure 1 and Table 1 show that the mapping precisions of all the methods increase as the number of markers increases, as expected.

**Figure 2:**
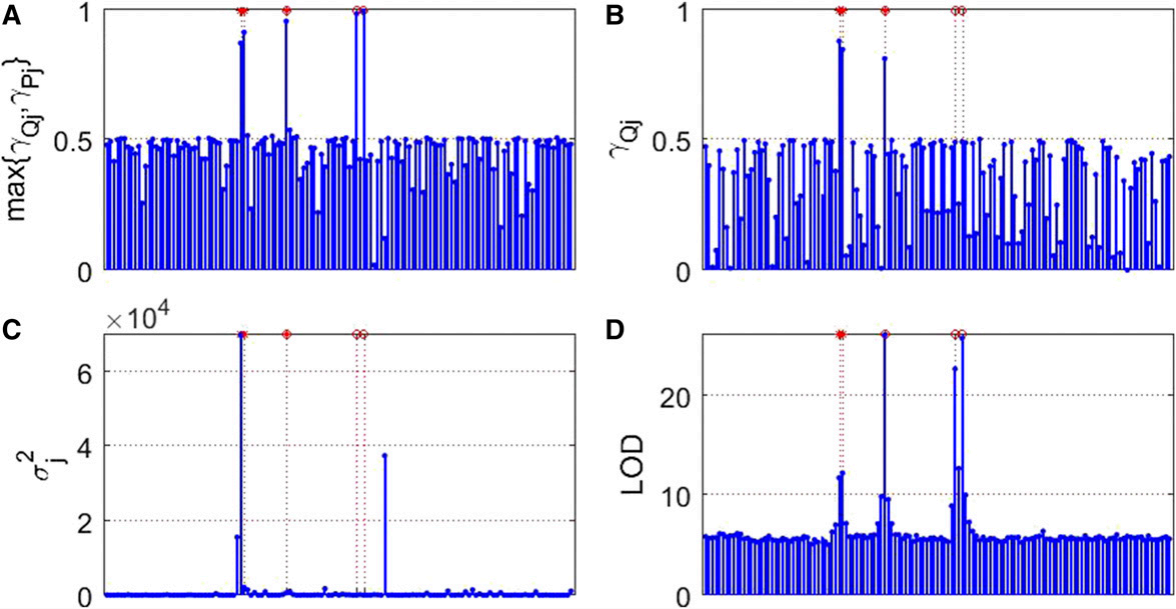
Estimate plot for case 1. *X*-axis: * indicates QTL location, and o indicates PoO location. *Y*-axis: (A) $\max\left\{\gamma_{Qj},\gamma_{Pj}\right\}$ (1 ≤ *j* ≤ *p*) in our proposed model (2); (B) $\gamma_{Qj}$ (1 ≤ *j* ≤ *p*) in the mixed model (3); (C) $\sigma_{j}^{2}$ (1 ≤ *j* ≤ *p*) in Yuan’s model (5); and (D) LOD scores for linear mixed-effects model (LMM) (4).

Figure 2 presents the Manhattan plots of the four models based on the average estimate of a total of 100 replications across the whole genome for case 1. Clearly, our proposed method is the winner in mapping genes with founder allelic effects (marked by an asterisk) and PoO effects (marked by a circle). The Manhattan plots for cases 2 and 3, and cases 1*, 2*, and 3*, are included in Figures S1–S5 in File S1, respectively, and show similar patterns.

## Discussion

The CC (Threadgill and Churchill 2012), a panel of newly emerged multiparent RI mouse strains, was developed, similar to other MPPs, to provide greater genetic diversity than the traditional RI populations and thereby to improve our power of understanding of complex traits. CC-RIX, F1 crosses that are generated from parental CC RI lines (Zou *et al.* 2005) can serve as excellent mouse models for mapping genes with both traditional genetic effects and epigenetic effects, such as imprinting. This study extends the model of Gong and Zou (2012) and Yuan *et al.* (2011) and, to the best of our knowledge, it is the first use of Bayesian variable selection methods to jointly map multiple QTL with both founder allelic effects and PoO effects for MPP-RIX data, in particular, CC-RIX data.

In this article, it is assumed that the RI lines are equally distanced from each other in terms of genetic distance which is a sensible assumption given the funnel design used for generating CC RI lines. However, we do observe that some RI lines share more or fewer founder alleles than expected. Our limited simulation shows that such genetic unbalance has a negligible effect on mapping genes, but this issue deserves further investigation. Alternatively, as genotypes of the parental RI lines are available, we could modify the design matrices $x_{j},$ $z_{j}$, and A in model (1) and replace them with local and genome-wide similarity matrices as done in sequence kernel association tests (SKAT) (Ionita-Laza *et al.* 2013) and genome-wide complex trait analysis (GCTA) (Yang *et al.* 2011). In addition, in our model we assume additive founder allelic effects. This assumption can be easily relaxed to allow genes with both additive and dominance effects (Woods *et al.* 2012; Zhang *et al.* 2014). Because dominant genetic effects are orthogonal to PoO effects, missing the dominant genetic effects does not cause any bias in the PoO effects estimate.

One of the drawbacks of Bayesian methods is their computational cost, especially for data with large numbers of samples and markers. Our model employs a block Gibbs sampler, which dramatically reduces computational time. However, further improvements may be possible. For example, instead of modeling each marker separately, we could jointly model multiple nearby markers and reduce the magnitude of *p*. Similar ideas have been proposed for genome-wide association study data (Wu *et al.* 2010; Lu *et al.* 2015). This approach is also biologically meaningful, since for some complex traits multiple causal SNPs may be located in a single region, and our simulations (cases 1*–3*) have shown that the proposed method is powerful in mapping regions with multiple causal SNPs. However, grouping nearby markers or SNPs may offer further help in improving mapping power, which deserves more thorough investigation.

## Supplementary Material

Supplemental material is available online at www.g3journal.org/lookup/suppl/doi:10.1534/g3.117.300483/-/DC1.
