# Supplementary material for: Joint Analysis of Strain and Parent-of-Origin Effects for Recombinant Inbred Intercrosses Generated from Multiparent Populations with the Collaborative Cross as an Example
Source: G3 (Bethesda). 2017 Dec 18;8(2):599–605. doi: 10.1534/g3.117.300483 (PMC5919741; doi:10.1534/g3.117.300483)
Supplement: Supplementary file 1 [file 599FileS1.docx]

**Supplementary Figure**
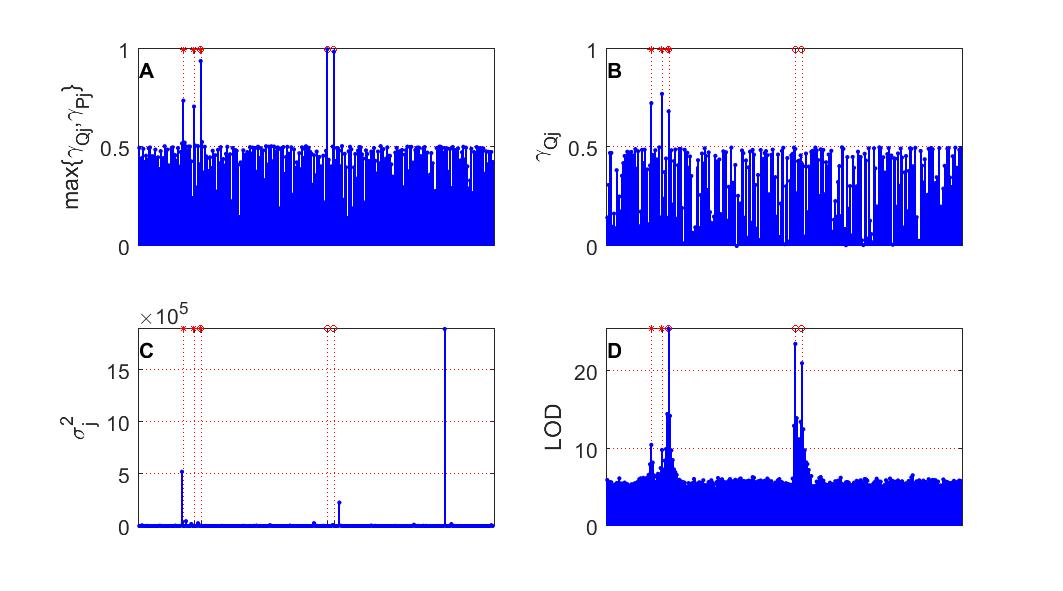


**Figure S1.** Estimate plot under case 2. X-axis: marker locations, star (*) indicates QTL location and circle (o) indicates PoO location. Y-axis: (**A**) $\max\left\{ \gamma_{Qj}, \gamma_{Pj} \right\}(1\leq j\leq p)$in our proposed model (2); (**B**) $\gamma_{Qj} (1\leq j\leq p)$ in Mixed model (3); (**C**) $\sigma_{j}^{2} (1\leq j\leq p)$ in Yuan's model (5) and (**D**) LOD scores for linear mixed effects model (LMM) (4).


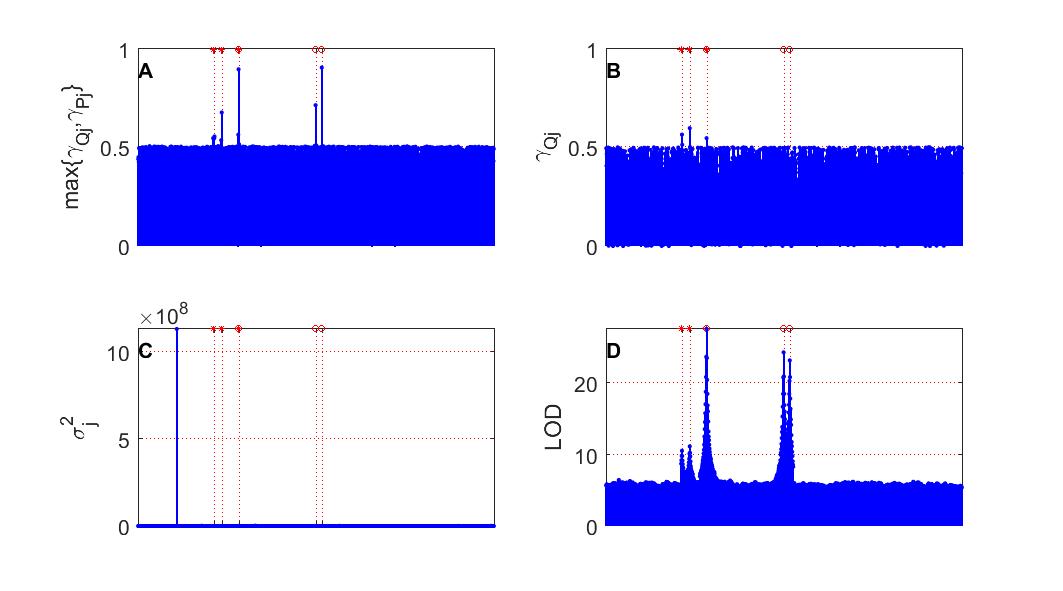
**Figure S2.** Estimate plot under case 3. X-axis: marker locations, star (*) indicates QTL location and circle (o) indicates PoO location. Y-axis: (**A**) $\max\left\{ \gamma_{Qj}, \gamma_{Pj} \right\}(1\leq j\leq p)$in our proposed model (2); (**B**) $\gamma_{Qj} (1\leq j\leq p)$ in Mixed model (3); (**C**) $\sigma_{j}^{2} (1\leq j\leq p)$ in Yuan's model (5) and (**D**) LOD scores for linear mixed effects model (LMM) (4).


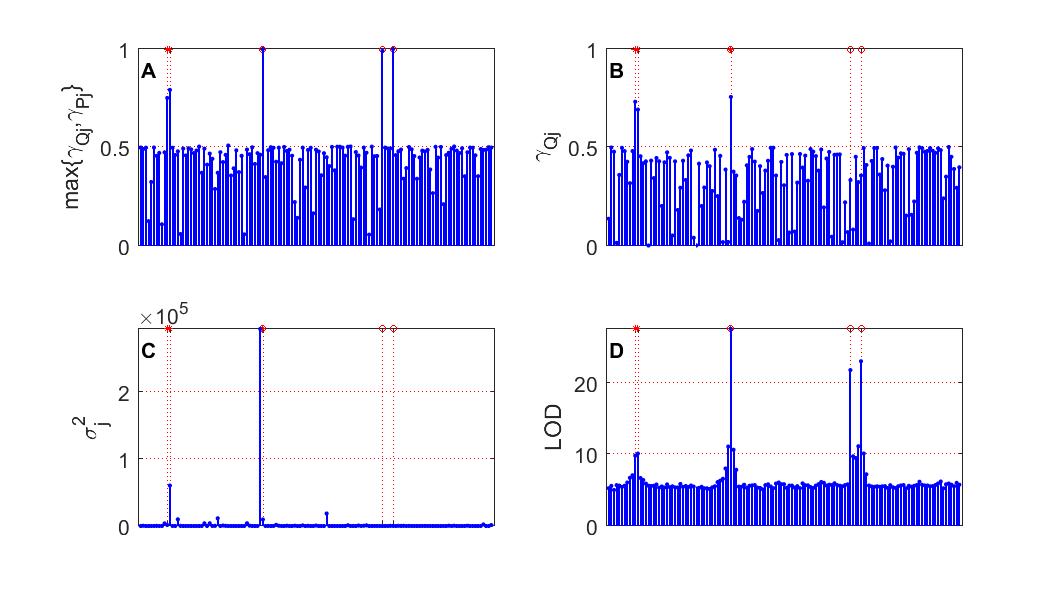
**Figure S3.** Estimate plot under case 1*. X-axis: marker locations, star (*) indicates QTL location and circle (o) indicates PoO location. Y-axis: (**A**) $\max\left\{ \gamma_{Qj}, \gamma_{Pj} \right\}(1\leq j\leq p)$in our proposed model (2); (**B**) $\gamma_{Qj} (1\leq j\leq p)$ in Mixed model (3); (**C**) $\sigma_{j}^{2} (1\leq j\leq p)$ in Yuan's model (5) and (**D**) LOD scores for linear mixed effects model (LMM) (4).


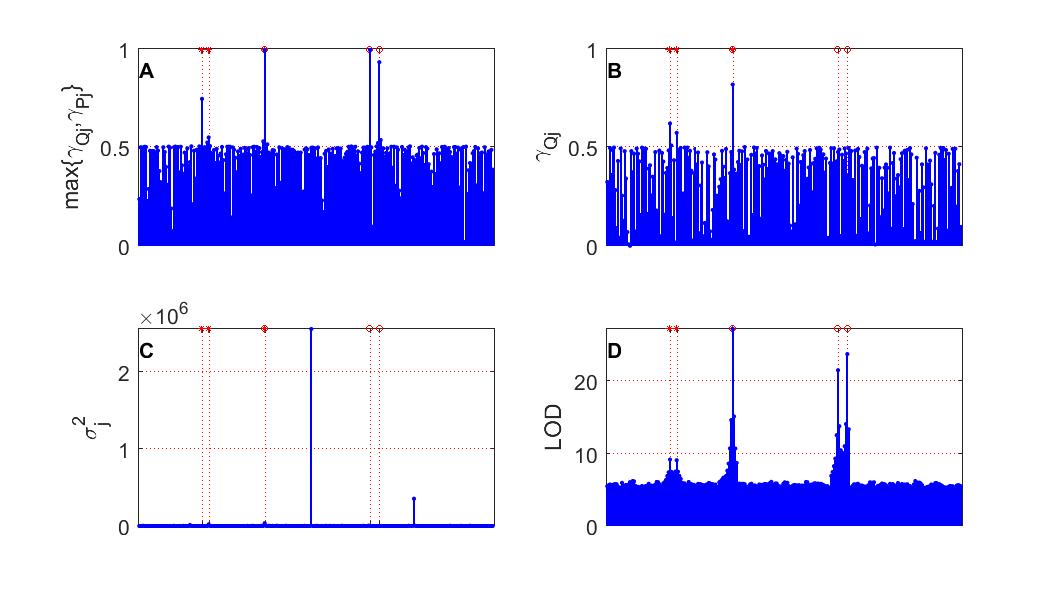
**Figure S4.** Estimate plot under case 2*. X-axis: marker locations, star (*) indicates QTL location and circle (o) indicates PoO location. Y-axis: (**A**) $\max\left\{ \gamma_{Qj}, \gamma_{Pj} \right\}(1\leq j\leq p)$in our proposed model (2); (**B**) $\gamma_{Qj} (1\leq j\leq p)$ in Mixed model (3); (**C**) $\sigma_{j}^{2} (1\leq j\leq p)$ in Yuan's model (5) and (**D**) LOD scores for linear mixed effects model (LMM) (4).


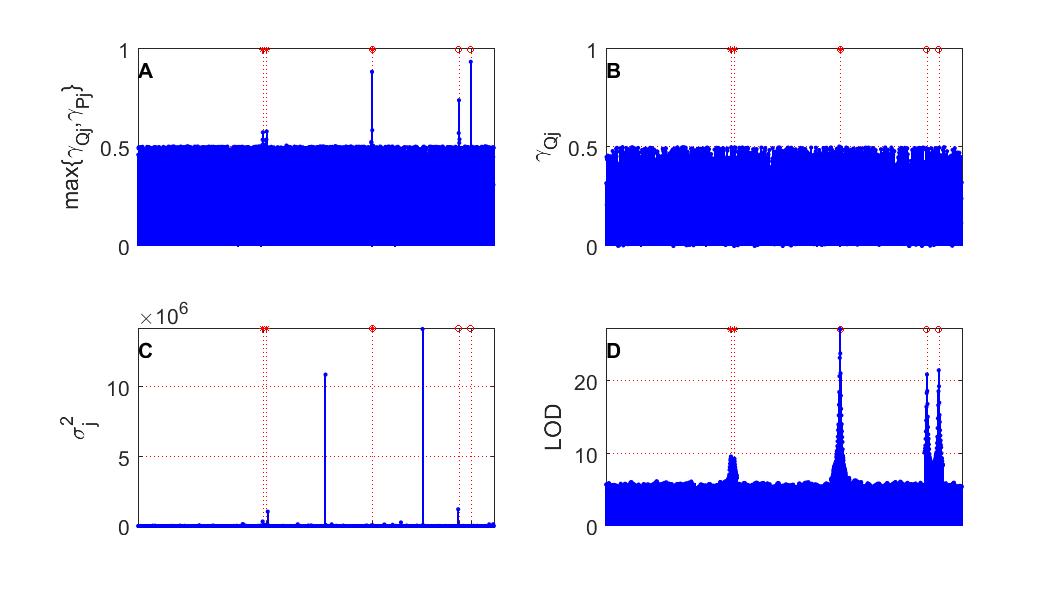


**Figure S5.** Estimate plot under case 3*. X-axis: marker locations, star (*) indicates QTL location and circle (o) indicates PoO location. Y-axis: (**A**) $\max\left\{ \gamma_{Qj}, \gamma_{Pj} \right\}(1\leq j\leq p)$in our proposed model (2); (**B**) $\gamma_{Qj} (1\leq j\leq p)$ in Mixed model (3); (**C**) $\sigma_{j}^{2} (1\leq j\leq p)$ in Yuan's model (5) and (**D**) LOD scores for linear mixed effects model (LMM) (4).
